# Rapid Antimicrobial Susceptibility Testing (RAST): A Quick Guide to Bacterial Sepsis Management

**DOI:** 10.7759/cureus.108766

**Published:** 2026-05-13

**Authors:** Sridevi Dinakaran, Sandhya Bhat, Shashikala Nair, Ravichandran Kandasamy

**Affiliations:** 1 Microbiology, Pondicherry Institute of Medical Sciences, Puducherry, IND; 2 Biostatistics, Pondicherry Institute of Medical Sciences, Puducherry, IND

**Keywords:** antimicrobial resistance, rapid antimicrobial susceptibility testing, sepsis, standard antimicrobial susceptibility testing, turnaround time

## Abstract

Background

Sepsis is a life-threatening condition that remains a major global health problem. Strengthening diagnostic stewardship is vital in combating sepsis. Administration of an antimicrobial agent based on a conventional antimicrobial susceptibility testing (AST) report requires about two days after a positive blood culture. In contrast, the European Committee on Antimicrobial Susceptibility Testing (EUCAST) Rapid Antimicrobial Susceptibility Testing (RAST) enables earlier antibiotic administration, reducing turnaround time (TAT) and improving survival. This study compares the reliability of RAST with standard AST (SAST) to establish RAST as a valuable diagnostic tool in our setting for managing bacterial sepsis.

Materials and methods

A prospective study (institutional ethics committee (IEC) number: RC/2023/53) was conducted in a tertiary care hospital. A total of 53 isolates were included in the study based on the inclusion criteria of monobacterial positive blood cultures. From a positively flagged blood culture bottle, Rapid Antimicrobial Susceptibility Testing (RAST) was performed, and the results of the eight-hour test were documented based on EUCAST RAST 2023 guidelines. Statistical analysis was performed using SPSS software version 29.0 (IBM Corp., Armonk, NY).

Results

Among the 53 isolates,* Escherichia coli* was the predominant isolate (49.1%, n=26), followed by *Klebsiella pneumoniae *(28.3%, n=15), *Pseudomonas aeruginosa *(11.3%, n=6), and *Acinetobacter baumannii *(11.3%, n=6). The average time to report by SAST was 47 hours and 45 minutes, while by RAST, it was 22 hours and 44 minutes. On comparison of the interpretative results of RAST with SAST, maximum agreement of 100% was documented for ceftazidime, ciprofloxacin, ceftazidime-avibactam, cefotaxime, and meropenem, with a kappa value of 1.000 and p-value of <0.001. We documented four minor errors (8%, n=4) in piperacillin-tazobactam, one minor error in amikacin (2%, n=1), and one major error in trimethoprim-sulfamethoxazole (2.12%, n=1). The kappa values for piperacillin-tazobactam, amikacin, and trimethoprim-sulfamethoxazole were 0.850, 0.956, and 0.957, respectively, with a p-value of <0.001.

Conclusion

RAST is of paramount importance in antibiotic stewardship, which has a significant impact on clinical decision-making in patients with sepsis.

## Introduction

Sepsis is a life-threatening condition with about 166 million cases and 21.4 million deaths reported globally in 2021 [1]. A study by Todi et al., called SEPSIS INDIA, tracked 1,172 patients with sepsis across 19 Indian ICUs for a year and found a mortality rate of 39.9% [2]. The Surviving Sepsis Campaign requires antibiotics to be given within one hour of diagnosis of sepsis, but the laboratory's current processes lead to prolonged turnaround time (TAT), delaying targeted therapy for patients on time [3].

Each hour of delay in antimicrobial therapy during the initial six hours independently reduces survival by an average of 7.6%, while excessive broad-spectrum antibiotic use drives multidrug resistance (MDR), affecting more than half of pathogens isolated from Indian ICUs [4,5]. Strengthening diagnostic stewardship is vital in combating sepsis.

Standard antimicrobial susceptibility testing (SAST) requires 24-48 hours after a positive blood culture for subculture, incubation, identification, and zone interpretation [6]. The European Committee on Antimicrobial Susceptibility Testing (EUCAST) Rapid AST (RAST) overcomes this limitation using direct disk diffusion from positive blood cultures at 4-8 hours, with predefined rapid breakpoints [7]. This enables earlier antibiotic administration, reducing turnaround time (TAT) and improving survival. European studies demonstrate 94%-98% categorical agreement with SAST, facilitating same-day de-escalation or escalation therapy optimization with standard laboratory infrastructure [8].

This study compares the reliability of RAST with standard AST (SAST) to establish RAST as a valuable diagnostic tool in our setting for managing bacterial sepsis.

## Materials and methods

A prospective study (IEC number: RC/2023/53) was conducted at the Pondicherry Institute of Medical Sciences, a tertiary care hospital. The study duration spanned from March 2023 to February 2024. Fifty-three isolates from monobacterial positive blood cultures were included, comprising only *Escherichia coli*,* Klebsiella pneumoniae*, *Pseudomonas aeruginosa*,and *Acinetobacter baumannii*, as RAST breakpoints were available only for these bacterial isolates.

From a positively flagged blood culture bottle, 125±25 µl of undiluted blood culture broth was pipetted onto each 90-mm circular Mueller-Hinton agar plate (BD BBL™ MHA II Agar). The broth was gently spread over the agar surface using a cotton swab in three directions. Antibiotic discs (Liofilchem) such as ceftazidime (10 µg), ciprofloxacin (5 µg), ceftazidime-avibactam (14 µg), cefotaxime (5 µg), meropenem (10 µg), amikacin (30 µg), piperacillin-tazobactam (36 µg), and trimethoprim-sulfamethoxazole (25 µg) were applied as per standard AST procedures, with four discs per plate employed to prevent interference between agents.

Plates were incubated at 37°C immediately, readings were taken at eight hours due to the readability of the zone of inhibition being better at this time, and results were documented according to EUCAST RAST 2023 guidelines [7]. Subsequently, standard AST (SAST) was performed and interpreted as per EUCAST 2023 guidelines [6]. The turnaround time (TAT) for the RAST report was calculated from the time the blood culture bottle was received until the eight-hour reading of the plates, whereas for SAST, it was calculated until the final authorization of the report in the laboratory information system (LIS).

Categorical agreement analysis was conducted by comparing the interpretive categories obtained from RAST and SAST, namely, susceptible (S), susceptible increased exposure (SIE), and resistant (R). Categorical agreement (CA) was defined as identical categorization between the two methods (S→S, R→R, SIE→SIE). Minor error (mE) was considered when RAST reported either susceptible (S) or resistant (R), while SAST categorized the isolate as susceptible increased exposure (SIE). Major error (ME) was defined as cases where RAST reported resistant (R), but SAST reported susceptible (S). Very major error (VME) was defined as cases where RAST reported susceptible (S), and SAST reported resistant (R) [9].

The area of technical uncertainty (ATU) is a range of inhibition zone diameters. It represents an area where the separation between susceptibility categories is poor. Interpretive errors increase dramatically in this area, and interpretation is not possible.

Statistical analysis was done using SPSS software version 29.0 (IBM Corp., Armonk, NY). Mean and standard deviation were used for continuous variables, while number and percentage were used for categorical variables. The agreement between the SAST and RAST methods was estimated using Cohen's kappa statistic. The average time taken to report by both methods was compared using a t-test. A p-value less than 0.05 was considered statistically significant.

## Results

The cohort comprised 30 male (56.6%) and 23 female (43.4%) patients. Patients exhibited a geriatric predominance, with 51.3% aged ≥51 years (mean age: 61.3±15.2 years). The peak incidence was in the 61-70 years group (32.1%, n=17), followed by the 51-60 years (24.5%, n=13). The monomicrobial positive blood cultures of 53 patients yielded *Escherichia coli* as the predominant pathogen (49.1%, n=26), followed by *Klebsiella pneumoniae* (28.3%, n=15), *Pseudomonas aeruginosa* (11.3%, n=6), and *Acinetobacter baumannii* (11.3%, n=6) (Table 1).

**Table 1:** Distribution of bacterial isolates from positive blood cultures

Organism	Number (n=53)	Percentage (%)
Escherichia coli	26	49.1
Klebsiella pneumoniae	15	28.3
Pseudomonas aeruginosa	6	11.3
Acinetobacter baumannii	6	11.3

**Figure 1:**
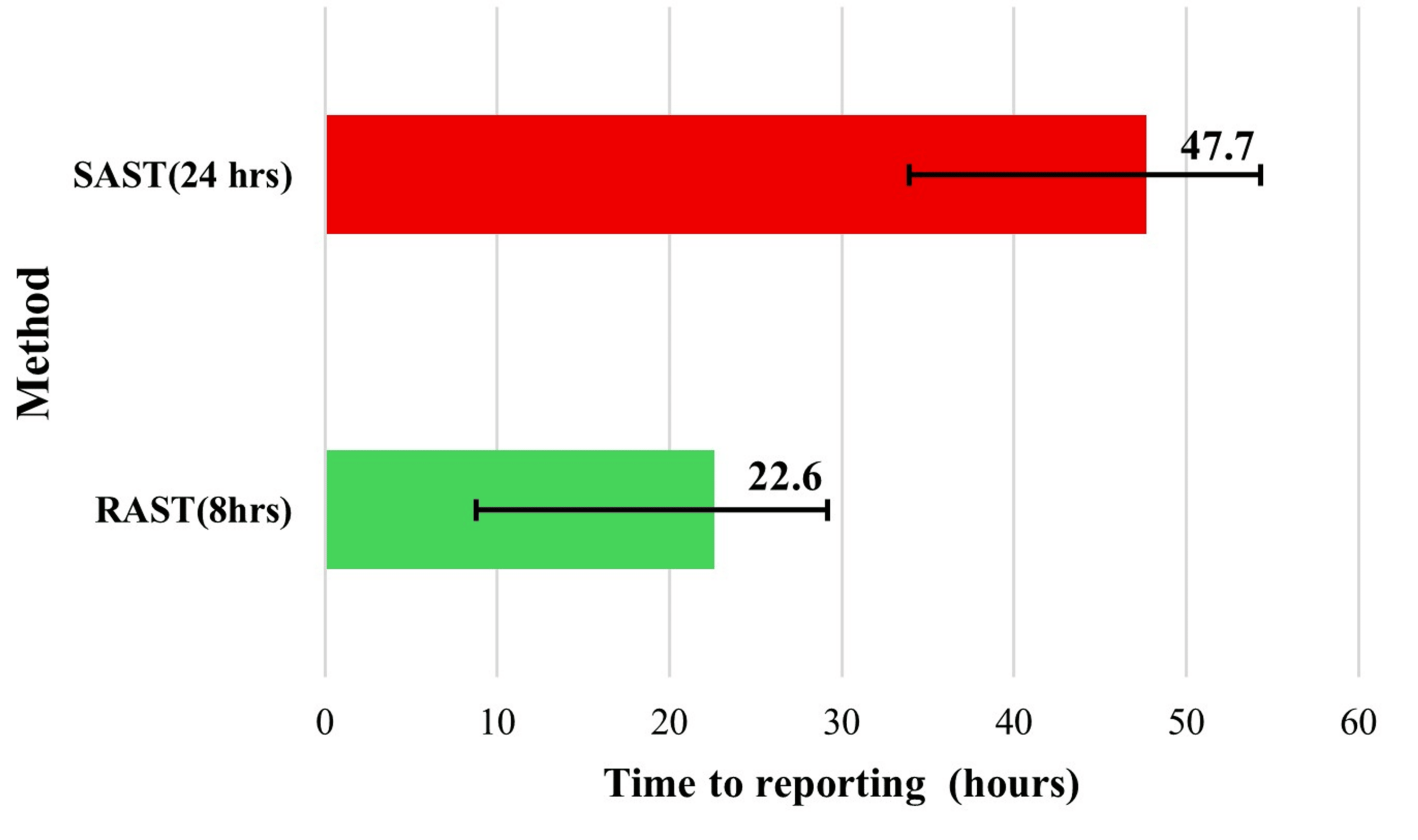
Turnaround time: RAST versus SAST RAST: Rapid Antimicrobial Susceptibility Testing, SAST: standard antimicrobial susceptibility testing

The average time to report by SAST is 47 hours and 45 minutes, while by RAST, it was 22 hours and 44 minutes. RAST dramatically reduced TAT by achieving a 52.6% reduction and saving a median of 22.5 hours (p<0.001) (Figure 1).

RAST and SAST interpretive categories were compared for eight antibiotics. RAST showed excellent concordance with five antibiotics (ceftazidime, ciprofloxacin, ceftazidime-avibactam, cefotaxime, and meropenem). Minor errors were seen with piperacillin-tazobactam (8%, n=4) and amikacin (2%, n=1). Trimethoprim-sulfamethoxazole (co-trimoxazole) had one very major error (2.1%, n=1). No major errors were reported (Table 2).

**Table 2:** Comparison of the interpretation results of the RAST and SAST methods n: number of isolates tested, RAST: Rapid Antimicrobial Susceptibility Testing, SAST: standard antimicrobial susceptibility testing

Antimicrobials	Minor error (%)	Major error (%)	Very major error (%)
Ceftazidime (n=50)	0 (0%)	0 (0%)	0 (0%)
Ciprofloxacin (n=53)	0 (0%)	0 (0%)	0 (0%)
Co-trimoxazole (n=47)	0 (0%)	0 (0%)	1 (2.12%)
Piperacillin-tazobactam (n=50)	4 (8%)	0 (0%)	0 (0%)
Ceftazidime-avibactam (n=50)	0 (0%)	0 (0%)	0 (0%)
Cefotaxime (n=41)	0 (0%)	0 (0%)	0 (0%)
Amikacin (n=50)	1 (2%)	0 (0%)	0 (0%)
Meropenem (n=53)	0 (0%)	0 (0%)	0 (0%)

Cohen's kappa analysis demonstrated 96% overall categorical agreement (κ=0.96) (p<0.001) for all tested antimicrobial agents. The five antibiotics (ceftazidime, ciprofloxacin, ceftazidime-avibactam, cefotaxime, and meropenem) achieved perfect concordance (κ=1.000) with immediate clinical utility. Near-perfect agreement was achieved for two antibiotics (co-trimoxazole and amikacin) (κ=0.96), while piperacillin-tazobactam showed clinically acceptable agreement (κ=0.850), warranting SAST confirmation. Absence of major errors confirms clinical safety (Table 3).

**Table 3:** Agreement and disagreement between the RAST and SAST methods RAST: Rapid Antimicrobial Susceptibility Testing, SAST: standard antimicrobial susceptibility testing

Antimicrobials	Total agreement	Total agreement (%)	Total disagreement (%)	Total disagreement (%)	Kappa value of agreement
Ceftazidime (n=50)	50	100	0	0	1.000
Ciprofloxacin (n=53)	53	100	0	0	1.000
Co-trimoxazole (n=47)	46	98	1	2.1	0.957
Piperacillin-tazobactam (n=50)	46	92	4	8	0.850
Ceftazidime-avibactam (n=50)	50	100	0	0	1.000
Cefotaxime (n=41)	41	100	0	0	1.000
Amikacin (n=50)	49	98	1	2	0.956
Meropenem (n=53)	53	100	0	0	1.000

## Discussion

Sepsis remains a major global health challenge, with prevalence especially high in low- and middle-income countries (LMICs) where access to care is limited [10,11]. Since every hour of delayed treatment worsens outcomes, RAST's rapid turnaround time is vital in sepsis management, where timely antibiotics can make all the difference. By providing quick results, RAST allows for earlier targeted therapy, which could improve survival and outcomes compared to conventional SAST, which often delays decisions [12].

In our study, the geriatric predominance (mean age: 61.3 years) is consistent with the SEPSIS INDIA registry (mean age: 57.4 years), highlighting RAST's value for elderly patients with comorbidities. The gram-negative predominance, led by *E. coli* (49.1%, n=26), reflects typical Indian ICU sepsis patterns [2].

RAST enables same-day adjustments for these high-risk pathogens, hitting that critical window before resistance worsens. RAST effectively closes the TAT gap in sepsis care, cutting reporting time from 47 hours and 45 minutes (SAST) to 22 hours and 44 minutes, a 52.6% reduction (p<0.001). This echoes European findings of 62.5% reductions versus SAST [13-15] and meets Surviving Sepsis Campaign goals for rapid diagnostics, outperforming traditional workflows [3].

Our results show RAST's robust performance (κ≥0.85 across agents), supporting routine use. It achieved perfect agreement for key drugs such as ceftazidime, meropenem, and ciprofloxacin, allowing fast adjustments in Indian ICUs.

Minor discrepancies appeared with piperacillin-tazobactam (8%, n=4) and amikacin (2%, n=1), but these were clinically acceptable and matched EUCAST RAST data: 4%-8% piperacillin-tazobactam errors from eight-hour zone variability and 2%-3.4% amikacin errors in Enterobacterales [16-18]. Co-trimoxazole discordance is likely due to slow trimethoprim diffusion at eight hours, consistent with the findings of Herroelen et al. of 16%-56% ATU at 4-8 hours [16].

Given co-trimoxazole's testing complexities, this very major error (VME) is acceptable. Since it is not a primary sepsis treatment choice, the preliminary report can be deferred and confirmed with SAST [19]. A result within the ATU is not interpretable; such results were reported as blank for the respective agent. Plates are re-incubated within 10 minutes after the 8-hour reading and re-read at 16-20 hours. If it is still within ATU, the complete result was not given, and SAST was performed.

This study confirms RAST as precise, reproducible, and reliable compared with SAST in our high-prevalence setting, offering local evidence for implementation.

India faces an antimicrobial resistance (AMR) crisis, but local RAST data are scarce. Showing ≥95% agreement can boost diagnostic stewardship, enable timely treatment adjustments, and reduce carbapenem overuse. These findings can help similar resource-limited countries to tackle sepsis mortality.

A limitation of this study was the small sample size and lack of patient outcome data, which constrained the evaluation of RAST's clinical impact.

## Conclusions

RAST plays a key role in antibiotic stewardship and really shapes how we manage sepsis cases. It delivers actionable results straight from positive blood cultures for gram-negative infections, letting us de-escalate therapy the same day, which is huge in high-burden places like India. Selective verification of piperacillin-tazobactam and co-trimoxazole results ensures patients' safety while optimizing antimicrobial stewardship efforts.
